# An evaluation of obstetrical data collection at health institutions in Mbarara Region, Uganda and Benue State, Nigeria

**DOI:** 10.11604/pamj.2024.47.109.36295

**Published:** 2024-03-06

**Authors:** Rajan Bola, Joseph Ngonzi, Fanan Ujoh, Raymond Bernard Kihumuro, Ronald Lett

**Affiliations:** 1 Canadian Network for International Surgery, Vancouver, Canada,; 2 Faculty of Medicine, Mbarara University of Science and Technology, Mbarara, Uganda,; 3 Center for Sustainability and Resilient Infrastructure and Communities, London South Bank University, London, United Kingdom

**Keywords:** Nigeria, obstetrical data, Uganda

## To the Editors of the Pan African Medical Journal

Obstetrical decision-making relies on comprehensive sources of accurate data. The breadth and completion rates of obstetrical data captured at health institutions in low-to-middle-income countries (LMICs) are not well documented. Previous studies based on maternal data collection and utilization of obstetrical guidelines in Benue State, Nigeria, and Mbarara Region, Uganda demonstrated major inconsistencies [1-3]. A minimal dataset for obstetrics is defined as a set of standardized measures used to index the minimum amount of data to obtain a global image of pregnant women across healthcare disciplines and in every stage of pregnancy [4]. Several minimal datasets have been developed for diseases and trauma registries in LMICs [5-7], however, no obstetrical minimal datasets have been proposed in the literature. Therefore, this assessment evaluated obstetrical data collection at health centers and hospitals in Mbarara, Uganda, and Benue State, Nigeria. This study was conducted at four health centers and one referral hospital in Benue State, Nigeria, and three health centers and one referral hospital in Mbarara Region, Uganda. We obtained all forms utilized to collect obstetrical data at health centers and hospitals for prenatal, perinatal, and postpartum records for up to 15 randomly sampled patients.

The percent completion for each variable captured in these forms was calculated. Variables were mapped to a minimal dataset that we proposed based on our previous work in Benue State; the Community Maternal Danger Score (CMDS). The CMDS is a validated 7-domain risk assessment tool that can predict the need for skilled maternal care [2]. It was validated in Benue State to predict maternal mortality with an accuracy of 85% and was based on pregnant women´s age, parity, patient size, obstetrical history, fundal height, coexisting conditions, and signs and symptoms of pre-eclampsia. The threshold for satisfactory data capture was chosen as 80% [7]. We examined the correlation between the number of collected variables and the average percent completion of these variables for each health center using Spearman´s correlation test [8]. Ethical approval was provided by the Ministry of Health and Human Services in Benue State, the Institutional Review Committee of Mbarara University of Science and Technology, and the Uganda National Council for Science and Technology. The number of captured variables ranged from 23 to 45 at Ugandan institutions, and 9 to 18 at Nigerian institutions. Table 1 illustrates the proportion of captured variables above the 80% threshold at health centers.

**Table 1:** description of health centers, number of variables recorded, number of CMDS variables and domains recorded, and completion rates

Health Center Name		Health Center Location	Total number of variables collected	Proportion of variables above 80% threshold	CMDS Domains collected at the health center	Number of CMDS variables collected	The proportion of CMDS variables above 80% threshold
Uganda	Kinoni Health Center	Mbarara	46	25/46 (54.3%)	All; age, parity, patient size, obstetrical history, fundal height, coexisting conditions, and signs and symptoms of pre-eclampsia	20	10/20 (50.0%)
	Mbarara City Health Center	Mbarara	46	23/46 (50.0%)	All; age, parity, patient size, obstetrical history, fundal height, coexisting conditions, and signs and symptoms of pre-eclampsia	21	9/21 (42.9%)
	Regional Referral Center	Mbarara	23	5/23 (21.7%)	3 of 7; age, obstetrical history, and signs and symptoms of pre-eclampsia	11	1/11 (9.1%)
	Mbarara Regional Referral Hospital	Mbarara	43	25/43 (58.1%)	All; age, parity, patient size, obstetrical history, fundal height, coexisting conditions, and signs and symptoms of pre-eclampsia	17	13/17 (76.5%)
Nigeria	Otukpo Primary Healthcare Center	Otukpo	18	15/18 (83.3%)	6 of 7; age, parity, patient size, fundal height, coexisting conditions, and signs and symptoms of pre-eclampsia	8	6/8 (75.0%)
	Gboko Primary Healthcare Center	Gboko	18	14/18 (77.8%)	6 of 7; age, parity, patient size, fundal height, coexisting conditions, and signs and symptoms of pre-eclampsia	11	10/11 (90.9%)
	Wadata Primary Healthcare Center	Wadata	11	4/11 (36.4%)	4 of 7; age, parity, obstetrical history, and signs and symptoms of pre-eclampsia	4	2/4 (50.0%)
	Family Support Programme	Makurdi	9	8/9 (88.9%)	3 of 7; age, obstetrical history, and signs and symptoms of pre-eclampsia	3	2/3 (66.7%)
	Federal Medical Center	Makurdi	11	11/11 (100%)	4 of 7; age, parity, obstetrical history, and signs and symptoms of pre-eclampsia	4	4/4 (100%)

CMDS; Community Maternal Danger Score

The CMDS was applied to assess the rates of completion for variables that predict pregnant women´s need for skilled birth care (Table 1). Within the Ugandan institutions, the 7 domains of the CMDS were adequately represented by the standard information recorded in clinical charts. In most centers, all 7 of the CMDS domains were reported for patients, with the Regional Referral Center being the only exception (3 of 7 domains represented). However, the majority of these variables were not consistently completed at the 80% threshold. The average proportion of CMDS variables that exceeded the 80% threshold from the 4 Ugandan institutions was 44.6% (range: 9-76%). Only one health center had greater than 50% completion rates: Mbarara Regional Referral Hospital. Within the Nigerian institutions, the 7 domains of the CMDS were not often represented within the standard set of information charted by care providers. Only two centers recorded information pertaining to 6 of 7 CMDS variables: Otukpo and Gboko Primary Healthcare Centers. However, the information that was routinely collected was more often complete. The average proportion of CMDS variables that exceeded the 80% threshold from the 5 Nigerian institutions was 76.5% (range: 50-100%). The correlation coefficient between the number of collected variables and the average percent completion of all variables was -0.33 (95% confidence interval: -0.41, 0.50; p=0.44).

Obstetrical data captured between and within institutions in Benue State and Mbarara are inconsistent. The Ugandan institutions captured many variables, but with lower completion rates. The Nigerian institutions collected fewer variables, but these were more complete. We were unable to compare the rates of obstetrical data capture with other countries due to a lack of this information in the literature. Almost all the domains of a minimal dataset defined by the CMDS are collected at Ugandan institutions. However, the Nigerian institutions lacked several of these vital variables. The correlation between the number of captured variables and completion above the 80% threshold was -0.33, indicating a trend of lower completion rates with more captured variables. However, the confidence intervals for this estimate were wide and did not indicate significance, possibly due to the small sample examined [9].

## Conclusion

These results demonstrate the importance of having a minimal, standardized dataset in LMIC settings where there may be limited time and resources to extract obstetrical data from charts with many variables. Rather, a minimal dataset would be more efficient, concise, and detailed, and therefore could be used to extract high-quality obstetrical information.
